# Fabrication of an Amperometric Flow-Injection Microfluidic Biosensor Based on Laccase for In Situ Determination of Phenolic Compounds

**DOI:** 10.1155/2015/845261

**Published:** 2015-10-05

**Authors:** Juan C. Gonzalez-Rivera, Johann F. Osma

**Affiliations:** CMUA, Department of Electrical and Electronics Engineering, University of Los Andes, Cra 1 E No. 19 A-40, Bogota, Colombia

## Abstract

We aim to develop an in situ microfluidic biosensor based on laccase from *Trametes pubescens* with flow-injection and amperometry as the transducer method. The enzyme was directly immobilized by potential step chronoamperometry, and the immobilization was studied using cyclic voltammetry and electrochemical impedance spectroscopy. The electrode response by amperometry was probed using ABTS and syringaldazine. A shift of interfacial electron transfer resistance and the electron transfer rate constant from 18.1 kΩ to 3.9 MΩ and 4.6 × 10^−2^ cm s^−1^ to 2.1 × 10^−4^ cm s^−1^, respectively, evidenced that laccase was immobilized on the electrode by the proposed method. We established the optimum operating conditions of temperature (55°C), pH (4.5), injection flow rate (200 *µ*L min^−1^), and applied potential (0.4 V). Finally, the microfluidic biosensor showed better lower limit of detection (0.149 *µ*M) and sensitivity (0.2341 nA *µ*M^−1^) for ABTS than previous laccase-based biosensors and the in situ operation capacity.

## 1. Introduction

Phenols are employed in several industries in the manufacture of plastics and plasticizers, resins, explosives, drugs, detergents, paper, fungicides, preservatives, dyes, and lubricants [1, 2]. Most phenolic compounds are toxic, noxious, and mutagenic and have carcinogenic activity [2] that accumulate in the environment and are found in food, potable water, sediments, and soil.

Currently, many organizations have established procedures using colorimetry, gas chromatography, liquid chromatography, capillary electrophoresis, and their variations [3]. Even though these methods attain accurate results for a wide range of phenolic compounds, conventional approaches are time-consuming and cost-intensive and require large volumes of organic solvents. Consequently, a market demand exists for a reliable, portable, simple, and cost-effective detection method of phenolic compounds.

Both enzymatic-based biosensors and microfluidic biosensors have attracted increasing among the different configurations of biosensors [4–8]. Microfluidic biosensors combine the advantages of fluidic microsystems, such as low cost, short analysis time, less consumption of sample and reagents, and portability, with the advantages of biosensors such as selectivity, moderate operational potentials, high sensitivity, specificity, and easiness to be miniaturized and integrated [3, 6, 9, 10]. Therefore they have potential in environmental safety, food, and clinic analysis.

The immobilization method is a key parameter for the design and fabrication of microfluidic biosensors [11]. The bioreceptor and the sensor elements can be coupled together with several methods, such as physical adsorption, entrapment, cross-linking, and covalent bonding [12, 13]. However, enzyme degradation and surface inaccessibility arise with the enzyme immobilization inside a microchannel. Thus, we propose the direct electrochemical immobilization of laccase after the sensor sealing since this technique enables an easier immobilization than traditional techniques.

Amperometry is the most common transducer technique in biosensors because it offers detection in real time [14, 15]. When this technique is coupled with convective mass transport, the resulting technique—hydrodynamic amperometry—offers additional assets such as increased current and sensitivity, quicker steady state, and smaller random contribution from natural convection [16]. Besides, the amount of analyte can be regulated directly by adjusting the flow rate of the flow injection system.

We aim to develop an amperometric flow-injection microfluidic biosensor based on laccase from* Trametes pubescens*. We studied the electrochemical immobilization of laccase by cyclic voltammetry and electrochemical impedance spectroscopy. The microfluidic biosensor electric response was evaluated with ABTS and syringaldazine, both well-known laccase substrates. The characterization of the biosensor included temperature, pH, flow injection, and applied potential effects on the signal response.

## 2. Materials and Methods

### 2.1. Reagents and Instrumentation

Glass slides of 76.2 mm in length and 25.4 mm in width were purchased at a local store. ABTS (2,2-azino-bis(3-ethylbenzothiazoline-6) sulphonic acid), syringaldazine, anhydrous ethanol, potassium ferricyanide, and potassium ferrocyanide were purchased from Sigma-Aldrich (USA). The developer (Microposit MF319) and positive photoresist (Microposit SC 1827) were purchased from Shipley (USA). Gold wire (Au, 99.99%) and chromium pieces (Cr, 99.95%) were purchased from Kurt J. Lesker (USA). PRS-100 positive photoresist stripper, dipotassium phosphate, sulfuric acid 97.8%, and hydrochloric acid 37.2% were purchased from J. T. Baker (USA). Potassium phosphate monobasic was purchased from AppliChem, (Germany); and nitric acid and hydrofluoric acid 40% were purchased from Panreac (Spain). PDMS was prepared according to product information from a Sylgard 184 silicone elastomer kit (Dow Corning, USA). All other chemicals used were of analytical degree.

Gold and chrome were deposited on microscopic slides using an Edwards Auto 306 thermal evaporation system at vacuum. The electrode pattern was transferred by an optical lithography maskless exposure system (model SF-100, Intelligent Micro Patterning, USA). The electrochemical procedures were measured using an Autolab Potensiostat/Galvanostat PGSTAT128N (Metrohm, USA) computer-controlled. Data were acquired and analyzed by the software Nova version 1.9. All assays were performed in a Faraday cage at room temperature.

### 2.2. Laccase Production

*T. pubescens* (CBS 696.94) was cultured on malt extract agar (MEA) plates during 10 days at 30°C. Cultures were carried out in 1000 mL shake flasks with 50 mL of basal medium and 15 g sterilized dry coffee husk [17]. Culture medium was inoculated with three 13 mm plugs from active fungus cultured in MEA. In brief, laccase was produced in 1 l shake flask with 50 mL of basal medium and 15 g sterilized coffee husk. Cultured medium was inoculated and incubated during 21 days at 30°C under static condition. The enzymatic crude extract was removed by filtration through 10 *μ*m filter paper (Boeco, Germany) and then centrifuged at 4°C and 4500 rpm for 15 min. Finally, the crude extract was filtered through 0.22 *μ*m Millex filter unit (Millipore, USA).

### 2.3. Free Laccase Activity Assay and Protein Measurement

The activity of free laccase was determined spectrophotometrically by measuring the absorbance change for 10 min using ABTS (*ε*_420_ = 36 mM^−1 ^cm^−1^) as a substrate at room temperature. To perform the assay, 950 *μ*L of 0.5 mM of ABTS in acetate buffer (0.1 M, pH 5.0) was mixed with 50 *μ*L of laccase crude extract. The enzyme activity was expressed as units per liter (U l^−1^), where U was defined as the amount of enzyme required to oxidized 1 *μ*mol ABTS per minute. Protein concentration was determined by Lowry assay [18].

### 2.4. Microfluidic Biosensor Fabrication

**Figure 1:**
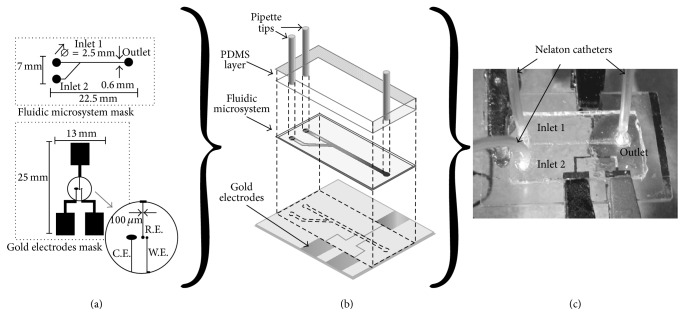
Masks and dimensions of the microfluidic biosensor (a); dismantled schematic diagram (b); and experimental set-up (c).

The device is composed of an upper glass slide with a fluidic microsystem and a lower glass slide with an integrated three-electrode arrangement made of gold (Figure 1(a)). Both pieces were fabricated through separate steps and assembled forward electrode modification with laccase. An in-channel configuration for the microfluidic biosensor was selected because it is the most preferred choice of amperometric microchip sensors [19]. The final device has a length of 35 mm and a width of 25 mm.

#### 2.4.1. Fabrication of the Gold Electrodes

The arrangement of gold electrodes was fabricated using first a chrome and latter a gold thin film deposition on glass slides by physical vapor deposition using a thermal evaporator Edwards E306 (Moorfield, UK) at 2.8 A with the metals on a tungsten slide at a vacuum pressure of 4 × 10^−5 ^mbar and an evaporation rate of 0.3 nm/min [20]. The chrome/gold deposition presented an overall thickness of 50 nm. The working electrode (WE) and reference electrode (RE) were fabricated with a diameter of 200 and 300 *μ*m, respectively. The counter electrode (CE) was fabricated with a transverse diameter of 1 mm and a conjugate diameter of 500 *μ*m.

Before the deposition of the gold layer, a chrome intermediate layer of 5 nm was evaporated to enhance the adhesion between the glass and gold. The electrode pattern was transferred over the metallic film by using mask-free optical lithography equipment with a resolution of 5 *μ*m. Positive photoresist was spin-coated onto the gold film at 3600 rpm for 60 s. Then, the photoresist was baked during 60 s at 115°C and patterned for 16 s. Finally, the soluble photoresist was removed submerging the substrate in the developer for 120 s with constant agitation.

The exposed gold areas were etched using aqua regia (HCl + HNO_3_ 3 : 1 v/v) for 5 s, and then the chrome was removed submerging the substrate for 30 s in HF 40% v/v. The residual baked photoresist was removed using stripper for 60 s leaving the three electrodes created on top of the glass slide (Figure 1(b) bottom).

#### 2.4.2. Fabrication of the Fluidic Microsystem

Copper foil tape was stuck on a glass slide, and the fluidic microsystem mask was transferred to the substrate by mask-free technology optical lithography, as was described for the electrode fabrication. Next, the copper pattern—obtained after the development—was exposed to FeCl_3_ 52% (w/v) with the aim to remove the unprotected copper and fabricate a physical mask for glass etching. Then, this slide with the pasted mask was submerged into an HF 40% solution for 4 min for glass etching. Therefore, a microchannel of 600 *μ*m of width and 250 *μ*m of depth was obtained in the glass slide. Finally, the copper mask was removed with FeCl_3_ 52% (w/v) leaving the glass fluidic microsystem clean of any sacrificial layers (Figure 1(b) middle).

The fluidic microsystem inlets and outlet were perforated in the glass fluidic microsystems using a commercial moto-tool (Figure 1(b) top). The holes were drilled—immersed in water—at 17,000 rpm using a diamond coated tip.

#### 2.4.3. Microfluidic Sensor Assembly

The fluidic microsystem and electrodes slides were assembled using UV curable epoxy. Previous to coating the fluidic microsystem slide with a glue layer, the microchannel was protected by filling it with positive photoresist to prevent channel clogging. After sealing both slides (Figure 1(b) bottom and middle) with UV curable epoxy, stripper was used to remove the positive photoresist from the microchannels.

Micropipette tips (0.2–10 *μ*L) were cut and used as connections between reservoirs and tubes. These tips were coupled in the inlets and outlet reservoirs of the fluidic microsystem using a layer of PDMS with a thickness of 2 mm (Figure 1(c)). The connectors were joined to Nelaton catheters that were coupled to the syringes.

#### 2.4.4. Laccase Immobilization

Laccase with an enzymatic activity of 2.13 U mg^−1^ of protein was selectively deposited on the working electrode inside the sealed fluidic microsystem by potential step chronoamperometry. To attain this process, a stream of crude extract of laccase (with a protein concentration of 0.178 mg mL^−1^) in 0.1 M phosphate buffer (pH 7.0) purged with high purity nitrogen was driven into the fluidic microsystem. Initial potential was set at the open-circuit potential. After 10 s of initial holding, laccase was immobilized by applying 1.2 V between the counter electrode and the working electrode for 3 min.

**Figure 2:**
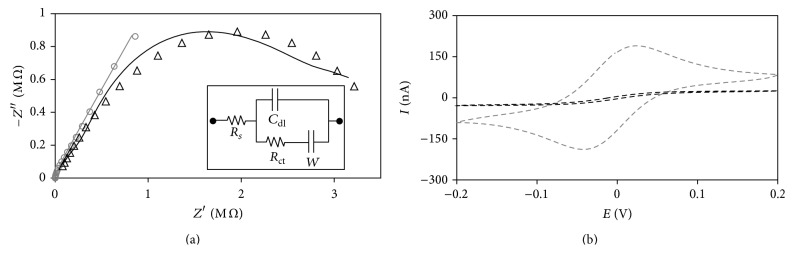
Nyquist plots (a) and cyclic voltammograms (b) at bare electrode (gray) and laccase modified Au electrode sealed in the fluidic microsystem (black). Continuous lines represent results from data fitting. Inset is the equivalent circuit. Solution: 1 mM K_4_[Fe(CN)_6_] + K_3_[Fe(CN)_6_] + 0.1 M phosphate buffer (pH. 7.0). For EIS, a sinusoidal potential modulation of ±5 mV was superimposed on the DC potential of 0.2 V versus Ag/AgCl/KCl_3M_. Applied frequency was from 10^6^ to 0.1 Hz. For CV the scan rate was 100 mV s^−1^.

Prior to use, the electrodes were electrochemically cleaned using a 0.1 M H_2_SO_4_ solution by successive cycling between −0.2 and 1.5 V at 500 mV s^−1^ until reproducible voltammograms were achieved (Figure 2(b)).

After laccase immobilization, 25 *μ*M ABTS in acetate buffer 0.1 M (pH 5.0.) was introduced in the sealed fluidic microsystems to test possible undesirable adsorption of laccase on the glass surface. No undesired laccase adsorption was detected through high magnification optical (>×1000) after several hours of ABTS exposure.

### 2.5. Electrochemical Study of Laccase Immobilization

Cyclic voltammetry (CV) and electrochemical impedance spectroscopy (EIS) were performed in 0.1 M phosphate buffer (pH 7.0) containing 1 mM Fe(CN)_6_^3−/4−^, due to the reasonably fast electron transfer. Voltammograms were scanned at 100 mV s^−1^ between −0.2 and 0.6 V. The frequency scan range for the EIS was from 0.1 Hz to 100 kHz and a sinusoidal potential modulation of ±5 mV was superimposed on the DC potential of 0.2 V. Before each experiment, fresh solution was purged for 10 min using high purity nitrogen. We selected the Randles circuit to fit the experimental data obtained by EIS; this circuit is comprised by a solution resistance (*R*_*s*_), a charge transfer resistance (*R*_ct_), a Warburg impedance (*W*), and a double layer capacitance (*C*_dl_). The diameter of the semicircle corresponds to the interfacial electron transfer resistance (*R*_ct_).

### 2.6. Characterization of the Microfluidic Biosensor Experimental Conditions

Temperature and pH effect on the immobilized laccase activity were studied by CV using ABTS as a substrate. Acetate buffer 0.1 M (pH 5.0) with ABTS 25 *μ*M was injected into the fluidic microsystem, and five voltammograms were scanned at 100 mV s^−1^ between −0.2 and 1 V. For each experiment the anodic peak height of the second voltammogram was plotted against the independent variable. Temperature effect was evaluated in the range from 20°C to 75°C at pH 5.0. Similarly, the buffer pH was varied from 4.0 to 7.5 at room temperature.

Injection flow rate effect on the immobilized laccase activity was evaluated by amperometry using acetate buffer 0.1 M (pH 5.0) as the running solution. The amperometry experiments with flow injection were conducted using the following procedure. The fluidic microsystem was treated with the running solution for 5 min. This solution was delivered into the fluidic microsystem using a syringe pump at a fixed flow rate. Then, ABTS 25 *μ*M in acetate buffer 0.1 M (pH 5.0.) was injected into the fluidic microsystem—through the inlet 2—using another syringe pump at the same rate of the running solution. The injection flow rate effect was evaluated at 100, 120, 140, 160, 180, 240, and 320 *μ*L min^−1^ applying 0.4 V. The current signal obtained was plotted against the independent variable. Each experiment was performed by triplicate. Data was plotted as relative current, which was defined regarding the maximum value of each experiment.

### 2.7. ABTS and Syringaldazine Detection Determination

ABTS and syringaldazine detection experiments were measured by amperometry with flow injection. The injection flow rate, buffer pH, and potential applied were chosen based on the results of the characterization of the experimental conditions. ABTS probes were performed with 0.1 M acetate buffer as the running buffer, and syringaldazine probes with 0.1 M acetate buffer-ethanol mixture prepared in a proportion 1 : 1 (v/v). Probes were made injecting continuously running buffer and the analyte was injected periodically each 120 s for 20 s increasing its concentration periodically. Experiments were conducted in triplicate and at room temperature unless specified.

We calculated the Michaelis-Menten parameters from the Lineweaver-Burk equation:

(1) $$\begin{array}{c} \frac{1}{I}=\frac{K_{M}^{\text{app}}}{I_{\max}\left[S\right]}+\frac{1}{I_{\max}}, \end{array}$$

where *I* is the current response, *K*_*M*_^app^ is the apparent Michaelis-Menten constant and *I*_max⁡_ is the maximum current measured under saturated substrate condition.

### 2.8. Stability of the Microfluidic Biosensor

The stability test followed the previous procedure for ABTS detection by amperometry with flow injection. Measurements were made each 24 h for 10 days (in triplicate at room temperature) using 0.1 M acetate buffer as the running buffer and 50 *μ*M ABTS in acetate buffer 0.1 M (pH 5.0) as the analyte solution for detection under the same conditions as for ABTS detection determination. The microfluidic biosensor was stored at 4°C after the measurements.

## 3. Results and Discussion

### 3.1. Laccase Immobilization

We determined the immobilization of laccase studying the electrochemical behavior of the working electrode. Previous the immobilization, the Nyquist profile shows a small semicircular profile at high frequencies, followed by a linear profile at low frequencies (Figure 2(a)). After the immobilization, the electrode only shows a semicircular profile within the frequency range evaluated. The estimated values of the interfacial electron transfer resistance (*R*_ct_) from the Randles model were 18.1 kΩ and 3.9 MΩ for the bare electrode and laccase-modified electrode, respectively.

We calculated the electron transfer rate constant (*k* ^0^, an indicator of the kinetic facility of the redox system) by EIS [21]. The following equations were applied for the 1-electron, first order reaction of the Fe(CN)_6_^3−/4−^ couple, assuming that *C*_ox_ = *C*_red_ = *C*, in order to determine *k* ^0^ [22]:

(2) RctRTFi0i0=FAk0C,

where *R* is the gas constant, *T* is the temperature, *F* is the Faraday constant, and *A* is the area of gold electrode. The *k* ^0^ measured were 4.6 × 10^−2 ^cm s^−1^ and 2.1 × 10^−4 ^cm s^−1^ for the bare and laccase-modified electrode, respectively.

Figure 2(b) shows the CV profiles obtained from the reaction of Fe(CN)_6_^3−/4−^ at the electrode interface before and after the immobilization of laccase. Fe(CN)_6_^3−/4−^ was used due to the reasonably fast electron transfer; however, by means of the modification of the charge transfer resistance and the electron transfer rate constant, the electrochemical immobilization of laccase on the electrode surface was evidenced. Previous the immobilization, the anodic and cathodic peak heights were 181.1 and 170.4 nA, respectively, and the peak separation was 54 mV. After the immobilization, the anodic and cathodic peak heights were 13.0 and 12.7 nA respectively, while the peak separation was 117 mV.

We induced the electrolysis of water applying a potential between the working and counter electrode in a buffer (pH 7.0) containing laccase. A decrease of the local pH in the vicinity of the working electrode produced the neutralization of laccase net charge (pI 2.6 for laccase from* Trametes pubescens* [23]). This process caused the precipitation of the enzyme on the surface of the working electrode [24, 25].

We found that laccase was immobilized on the electrode sealed inside the fluidic microsystem by this electrochemical technique without exposing laccase to denaturing conditions. The change of the Nyquist profiles showed that the Fe(CN)_6_^3−/4−^ reaction was initially limited by the mass transfer of the active specie from the bulk solution to the electrode interface, but the reaction shifted to kinetic-limited after the immobilization [16]. This change is evidenced by the decrease of two orders of magnitude of *k* ^0^ and the increase of *R*_ct_. This behavior means that the Fe(CN)_6_^3−/4−^ reaction was harder to accomplish due to the presence of a layer of laccase on the surface of the working electrode, which decreased the active area of the electrode. After the immobilization, we also observed a decrease of an order of magnitude in the current response and an increase of 54% in the potential separation of the peaks by CV. These observations proved that the reaction—initially reversible—became irreversible with the functionalization of the electrode. These observations proved the hypothesis that laccase was immobilized by the electrochemical technique performed in this work.

### 3.2. Characterization of Experimental Conditions of the Microfluidic Biosensor

**Figure 3:**
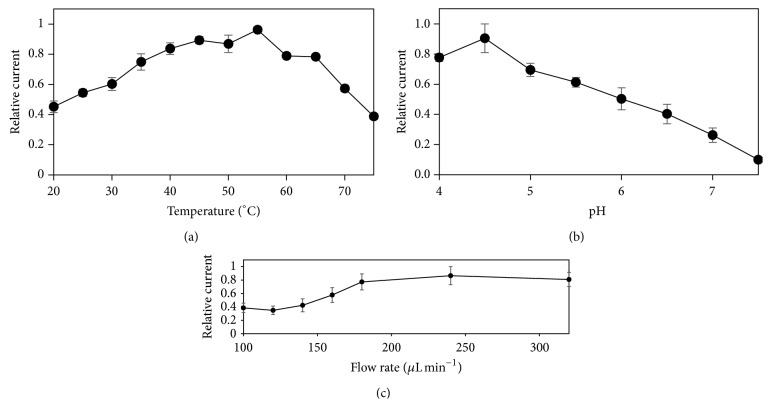
Effect of temperature (a), pH (b), and injection flow rate (c) on the current response of the microfluidic biosensor. Error bars describe the standard deviation of the three replicates. Solution: acetate buffer 0.1 M (pH 5.0.) as the running solution and ABTS 25 *μ*M in acetate buffer 0.1 M (pH 5.0.) as the analyte.

Figure 3 shows the resulting relative current from the ABTS oxidation by the working electrode modified with laccase when we evaluated the effect of temperature, pH, injection flow rate, and potential applied on the current response. The temperature profile achieved a maximum around 55°C, while the pH profile showed a maximum around 4.5 (Figures 3(a) and 3(b)). We found previously using free laccase from* Trametes pubescens* and ABTS as a substrate that the temperature profile behaved like a bell-shape with a maximum around 55°C, and the pH profile decreased as the pH increased, with a maximum in a range from 2 to 3 [26]. These profiles behaved similarly compared to the profiles from the present work, which may indicate that the electrochemical immobilization proceeded without modify the structure of the enzyme.

The maximum current was achieved at a temperature higher than previous laccase-based biosensors using nanomaterial composites and polymers [27–29] and similar to laccase immobilized on magnetic chitosan microparticles (55°C) [30]. Also, the behavior of pH profile is consistent with previous biosensors characterizations [31, 32].

Figure 3(c) shows that the current increases with the flow rate from 100 to 180 *μ*L min^−1^, and after the turning point around 180 *μ*L min^−1^ the current became constant. Before the turning point, heterogeneous mass transfer kinetic is higher than the rates of mass transfer; therefore the reaction is kinetic controlled. After the turning point, the convective contribution to mass transfer became larger to increase the mass transfer rates turning to a mass-transfer controlled reaction. Based on these results, we selected a temperature of 55°C, a buffer pH of 4.5, an injection flow rate of 200 *μ*L min^−1^, and a potential applied of 0.4 V as the optimal conditions for phenolic determination.

### 3.3. Detection of ABTS

**Figure 4:**
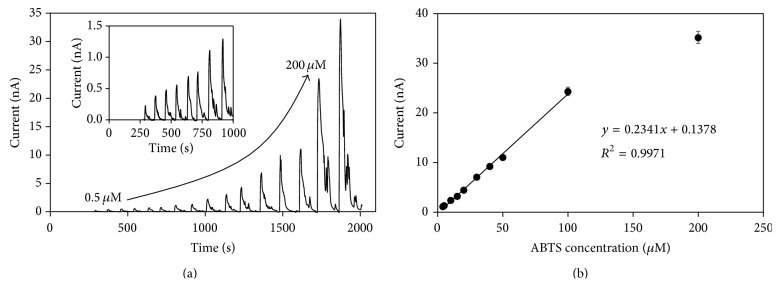
Current-time response curve with background signal subtracted (a) and calibration curve (b) of the microfluidic biosensor. Increasing concentrations of ABTS (from 0.5 *μ*m to 200 *μ*M) in 0.1 M acetate buffer (pH 4.5) were injected at 200 *μ*L min^−1^. Potential applied of 0.4 V at room temperature. Error bars describe the standard deviation of the three replicates.

Figure 4 shows the current response achieved at different concentrations of ABTS. We achieved a linear relationship within 0.5 and 100 *μ*M with a relative standard deviation (RSD) lower than 5%. The ABTS sensitivity and the detection limit (signal-to-noise ratio (S/N) = 3) calculated were 0.2341 nA *μ*M^−1^ (741 nA *μ*M^−1 ^cm^−2^) and 0.149 *μ*M, respectively. The apparent Michaelis-Menten constant (*K*_*M*_^app^) and the maximum current (*I*_max⁡_) were 386.5 *μ*M and 105 nA, respectively. The sampling rate of the microfluidic biosensor calculated was 24 to 60 samples per min.

The substrate sensitivity was improved compared with the laccase electrode covalently immobilized on platinum and platinum oxide (75 nA *μ*M^−1^) [31, 33] and laccase on glassy carbon electrodes (358.3 ± 18.8 nA *μ*M^−1 ^cm^−2^) [34]. The detection limit was also lower than those reported in previous results for amperometric laccase biosensors on platinum (0.2 *μ*M) and platinum oxide (0.5 *μ*M) [31, 33], as well as the laccase biosensor based on a matrix of carbon nanotubes-chitosan composite (0.23 *μ*M) [32].

**Figure 5:**
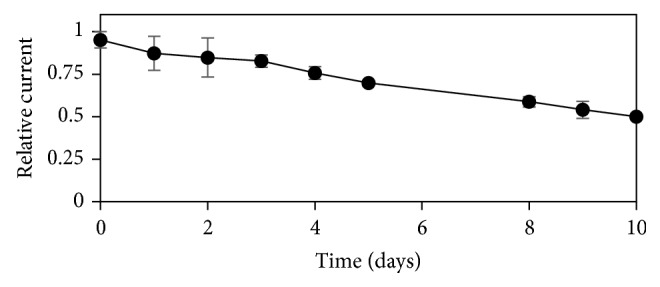
Stability of the microfluidic biosensor. Measurements made each 24 h for 10 days using 0.1 M acetate buffer as the running buffer and 50 *μ*M ABTS in acetate buffer 0.1 M (pH 5.0.) as the analyte solution for detection. Error bars describe the standard deviation of the three replicates.

Figure 5 shows the stability of the microfluidic biosensor. This test showed a linear decrease with a coefficient of determination (*R* ^2^) of 0.9924, and a half-life time of 10 days. The relative standard deviation was lower than 10%.

### 3.4. Detection of Syringaldazine

**Figure 6:**
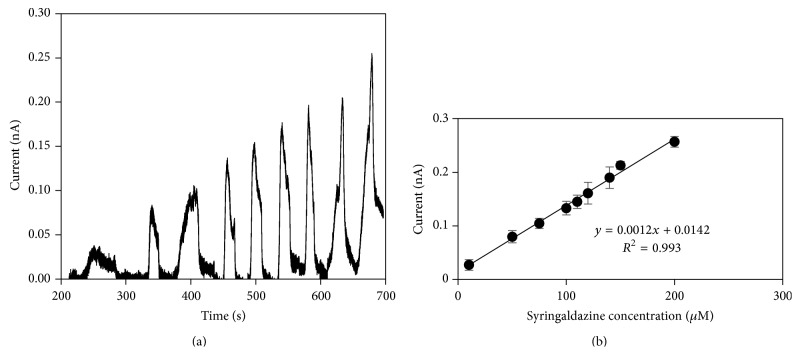
Current-time response curve with background signal subtracted (a) and calibration curve (b) of the microfluidic biosensor. Increasing concentrations of syringaldazine (from 10 *μ*m to 200 *μ*M) in 0.1 M acetate buffer (pH 4.5) : ethanol 1 : 1 (v/v) solution were injected at 200 *μ*L min^−1^. Potential applied of 0.4 V at room temperature. Error bars describe the standard deviation of the three replicates.

Figure 6 shows the current response achieved at different concentrations of syringaldazine; we achieved a linear relationship within 10 and 200 *μ*M with a relative standard deviation (RSD) lower than 10%. The syringaldazine sensitivity and the lowest detectable concentration were 0.0012 nA *μ*M^−1^ and 10 *μ*M, respectively. The apparent Michaelis-Menten constant (*K*_*M*_^app^) and the maximum current (*I*_max⁡_) were 540 *μ*M and 0.9 nA, respectively. Also, the microfluidic biosensor is capable of measure 60 syringaldazine samples per min.

We found that the sensitivity and the repeatability for syringaldazine decreased compared with ABTS values. This biosensor has a ABTS limit of detection comparable to the biosensor of laccase covalently immobilized on a composite of silver nanoparticles, carboxylated multiwalled carbon nanotubes and polyaniline on a gold surface [35], a laccase biosensor based on platinum nanoparticles dispersed in 1-butyl-3-methylimidazolium hexafluorophosphate [36], and a laccase based biosensor immobilized on magnetic core-shell nanoparticles, but lower for the detection of syringaldazine [37]. Apparently ethanol, which has a lower polarity than water (relative permittivity: *ε*_*r*H2O_ = 78.5; *ε*_*r*C2H6O_ = 24.5 at 25°C), affected the performance of the biosensor; as polarity promotes the dissociation of dissolved electrolytes and hydration of the ions, the ion mobility was much harder [38]. However, this should not be a problem in real sample since ethanol has a high volatility, and phenolic compounds can be found in aqueous solutions.

## 4. Conclusions

The complete process of fabrication, assembling, and enzymatic immobilization of a microfluidics biosensor for the detection of phenol is described. This microfluidics system can be functionalized after the microfabrication process takes place and the microdevice is sealed and operates in both stationary and continuous flow conditions. The material of the substrate and the confinement of the electrodes allow this microdevice to be operated in situ without any risk of deterioration or contamination of the sample. In addition, a small volume is needed to detect phenol in aqueous solutions thanks to the microchannel structure. The microfluidic biosensor showed better analytic characteristics than previous biosensors, such as the lower limit of detection and sensitivity. Moreover, the optimum operational conditions of temperature, pH, injection flow rate, and potential were established and can be directly applied to in situ operation as well as its fabrication procedure introduced for industrial applications.

## Abbreviation

ABTS: 2,2-Azino-bis(3-ethylbenzothiazoline-6) sulphonic acid
ASTM: International Association of Testing Materials
CE: Counter electrode
CV: Cyclic voltammetry
EIS: Electrochemical impedance spectroscopy
EPA: Environmental Protection Agency
ISO: International Organization for Standardization
RE: Reference electrode
RSD: Relative standard deviation
WE: Working electrode.
